# Commentary: Exploring Mental Health Status and Syndrome Patterns Among Young Refugee Children in Germany

**DOI:** 10.3389/fpsyt.2019.00341

**Published:** 2019-05-10

**Authors:** Alameen Damer, Talha Tahir, Michael Wong

**Affiliations:** ^1^Bachelor of Health Sciences Program, McMaster University, Hamilton, ON, Canada; ^2^Psychology Department, University of Wisconsin–La Crosse, La Crosse, WI, United States

**Keywords:** refugee, mental health, child psychopathology, Child Behavior Checklist, Caregiver Teacher Report Form

## Introduction

The worldwide refugee crisis has given rise to 16.2 million refugees in 2017 alone, 52% of which were children (1); yet, there is a dearth of information on the mental health status of these children. Buchmüller et al. (2) analyzed mental health patterns in Syrian and Iraqi refugee children aged 1.5–5 years, who recently arrived in Germany. Two studies were conducted: the first used the Child Behavior Checklist (CBCL) to examine child mental health as reported by a parent; the second, conducted on a separate sample, used the Caregiver Teacher Report Form (CTRF) to assess child mental health as reported by teachers. When compared to clinical reference data of U.S. children, refugee parents reported elevated levels of anxiety/depression, attention problems, and withdrawal behavior in their children. Reports by teachers showed elevated levels of mental health problems in refugee children when compared to U.S. norms, but not when compared to clinical reference data. Therefore, the authors of the study were unable to elucidate a clear refugee-specific mental health pattern among preschool children (2). We discuss here potential factors contributing to the inconsistent results reported by Buchmüller et al. (2).

## Maternal Psychopathology

Buchmüller et al. (2) did not account for the potential effects of maternal psychopathology, which may explain the discrepancy observed between the CBCL and CTRF scores. Refugees often experience traumatic events (3). A study by East et al. (4) investigating Somalian refugee women found that, on average, each woman had experienced 24 traumatic events. Additionally, refugees resettled in western countries are 10 times more likely to have posttraumatic stress disorder than the general population (5). Given that respondents in part one of Buchmüller et al.’s (2) study were refugees, it is plausible that there was an increased prevalence of psychopathology in this sample. Mothers with psychopathology often focus on negative events, recalling them more easily than positive ones, possibly distorting the results of the CBCL (6). Carneiro et al. (6) also found that problem scores on the CBCL were positively associated with maternal psychopathology. Moreover, disagreement between symptoms reported by mothers *via* the CBCL and symptoms reported by teachers *via* the CTRF was positively associated with maternal psychopathology (6). Buchmüller et al. (2) found mental health status on the scales of anxious/depressed, withdrawal, and attention problems to be in the clinical range on the CBCL as reported by parents. However, these findings were not corroborated by the results of the CTRF, which reported subclinical levels in these three scales. There is a possibility that the discrepancy seen between the CBCL and CTRF scores is the result of the unadjusted effects of maternal psychopathology. It is noteworthy to mention that Buchmüller et al. only included one child per family but did not report birth order of the children assessed. There is some suggestion from the literature that birth order has differential effects on psychopathology, which may in turn affect the CBCL scores reported (7–9).

## Disagreement between CBCL and CTRF

The CBCL and CTRF consist of 100 questions assessing child behavior on eight scales; these scales are organized into three categories: internalizing problems, externalizing problems, and total problems (10). Buchmüller et al.’s (2) use of both the CBCL and CTRF led to inconsistent results regarding the existence of a refugee-specific mental health pattern. According to the literature, there appears to be a low-moderate agreement between the CBCL and CTRF (11). A meta-analysis by Huang (11) found the correlation between parent and teacher ratings to range from 0.18 to 0.35. Furthermore, Rescorla et al. (12) found that scores on CBCL were significantly higher than scores on the CTRF on most problem scales across 21 societies. This point parallels the results of Buchmüller et al. (2) in which CBCL scores were higher than CTRF scores across most problem scales. Additionally, Rescorla et al. (12) found that a deviant score on the CBCL was only corroborated by a deviant score on the CTRF a third of the time. These findings provide a possible explanation for why clinical levels of anxiety/depression, attention problems, and withdrawal behavior reported by parents were not corroborated by teachers in Buchmüller et al.’s (2) study. The design of Buchmüller et al.’s (2) study perhaps also played a role in the discrepancy observed between CBCL and CTRF scores. Given that the CBCL and CTRF were filled out for different samples of children, it is difficult to directly examine the agreement between the tests. There exists the possibility that the scores differed simply because the two samples had different levels of psychopathology.

## Parental Education

The sample of parents used by Buchmüller et al. (2) had a mean education time of 10.3 years, which is lower than the mean education of 13.4 years reported in the U.S. female populace in 2016 (13). The comparative undereducation of the refugee parents may have affected their ability to accurately complete the CBCL (6). A study by Tehrani-Doost et al. (14) reported that higher maternal education levels were significantly predictive of lower scores on many categories of the CBCL. Carneiro et al. (6) also found a similar negative correlation between maternal education and problem scores on the CBCL. This is possibly because more educated mothers are better informed of typical emotional and behavioral development in children and are less likely to rate normative behavior as problematic (14). In Buchmüller et al.’s (2) study, the mothers’ relative undereducation may have increased the effect size for multiple subscales of the CBCL, overreporting problematic behavior, and potentially misrepresenting a mental health pattern.

## Conclusion

Buchmüller et al.’s (2) study reported elevated CBCL problem scores among refugee children, a finding that may have been the product of maternal psychopathology and low education present within the parental sample. Moreover, the disparity observed between the CBCL and CTRF scores may simply be the result of intrinsic disagreement between the two instruments instead of measurable differences in psychopathology. Despite these potential limitations, studies of this nature are nevertheless important to analyze mental health within child refugee populations and meet their needs with tailor-made clinical interventions.

## Author Contributions

All authors listed have made a substantial, direct, and intellectual contribution to the work, and approved it for publication.

## Conflict of Interest Statement

The authors declare that this manuscript was written in the absence of any commercial or financial relationships that could be construed as a potential conflict of interest.
